# Impact of baseline glucocorticoid use on the efficacy of immunotherapy combined with intracranial radiotherapy in NSCLC patients with brain metastases

**DOI:** 10.1093/noajnl/vdaf158

**Published:** 2025-07-12

**Authors:** Jin Xiong, Wenhao Shi, Yusheng Huang, Hongmei Jian, Zaicheng Xu, Hongjun Tang, Shunping Huang, Zhenzhou Yang, Yuan Peng

**Affiliations:** Department of Cancer Center, The Second Affiliated Hospital, Chongqing Medical University, Chongqing, 400010, China; Department of Cancer Center, The Second Affiliated Hospital, Chongqing Medical University, Chongqing, 400010, China; Department of Cancer Center, The Second Affiliated Hospital, Chongqing Medical University, Chongqing, 400010, China; Department of Cancer Center, The Second Affiliated Hospital, Chongqing Medical University, Chongqing, 400010, China; Department of Cancer Center, The Second Affiliated Hospital, Chongqing Medical University, Chongqing, 400010, China; Department of Cancer Center, The Second Affiliated Hospital, Chongqing Medical University, Chongqing, 400010, China; Department of Cancer Center, The Second Affiliated Hospital, Chongqing Medical University, Chongqing, 400010, China; Chongqing key laboratory of tumor immunotherapy, Chongqing, 400037, China; Department of Cancer Center, The Second Affiliated Hospital, Chongqing Medical University, Chongqing, 400010, China; Chongqing key laboratory of tumor immunotherapy, Chongqing, 400037, China; Guchengtai Community Health Center of Chengxi District, Xining, 810000, China; Department of Cancer Center, The Second Affiliated Hospital, Chongqing Medical University, Chongqing, 400010, China

**Keywords:** brain edema, glucocorticoids, immunotherapy combined with intracranial radiotherapy, NSCLC brain metastases

## Abstract

**Background:**

Glucocorticoids (GCs) play a crucial therapeutic role in managing cerebral edema caused by radiation-induced acute brain injury or brain metastases. However, they negatively impact the efficacy of immunotherapy.

**Methods:**

We collected data from 62 patients with non-small cell lung cancer (NSCLC) brain metastases who received immunotherapy combined with intracranial radiotherapy within 28 days of each other. The overall doses of GCs for each patient were expressed as the cumulative total dose of dexamethasone equivalents during the baseline period of immunotherapy. These patients were categorized based on their baseline GCs usage (28 days before and after the first immunotherapy) into three groups: low GCs use (< 30 mg), medium GCs use (30–100 mg), and high GCs use (≥ 100 mg).

**Results:**

Among the three groups of included patients in our study, the median intracranial progression-free survival (iPFS) was significantly shorter in the high GCs use group compared to the medium and low GCs use groups (5.23 months vs. 12.70 months vs. 16.43 months, *P* < .001). No significant difference in median iPFS was observed between the medium and low GCs use groups. Median overall survival (OS) and median progression-free survival (PFS) among the three groups had a similar trend with iPFS. No significant differences in intracranial objective response rate (iORR) and objective response rate (ORR) were found among the three groups. Standard propensity score matching (PSM) confirmed that the high GCs use group (≥ 100 mg) still had significantly shorter median iPFS, PFS, and OS compared to the other groups.

**Conclusions:**

Increased baseline GCs use is associated with reduced efficacy of the combination therapy in these patients and baseline high-dose GCs use (≥ 100 mg) notably impairs survival outcomes.

**Key Points**

1. Increased baseline GCs intake associates with reduced efficacy of the combined immunotherapy and intracranial radiotherapy.
2. Baseline high-dose GCs use (≥ 100 mg) notably impairs survival outcomes of NSCLC patients with brain metastasis.

**Importance of the Study**

This study revealed that increased baseline GCs use is associated with reduced efficacy of the combination therapy in these patients and baseline high-dose GCs use (≥ 100 mg) notably impairs survival outcomes. Therefore, optimizing the use of anti-edema drugs, such as mannitol, bevacizumab, diuretics, or hypertonic saline in combination with brain radiotherapy and immunotherapy could be considered to mitigate the adverse effects of GCs on the efficacy of ICIs. This discovery could be applied in the clinical practice for these patients in the future.

Brain metastases occur in approximately 10%–20% of patients with advanced NSCLC at the time of diagnosis and develop in 20%–40% of these patients during disease progression.^1^ Targeted therapies have proven highly effective for patients with driver-positive gene mutations in NSCLC brain metastases and are widely used.^2^ For patients without driver-gene mutations, intracranial radiotherapy combining with systematic therapy is a commonly applied option in clinical practice. Numerous retrospective and evidence-based medical studies have confirmed that the combined use of immunotherapy provided superior therapeutic effects on intracranial lesions comparing to standalone brain radiotherapy.^3–5^ Immunotherapy and radiotherapy play synergistic effect in inhibiting tumor progression. Radiotherapy induces and enhances tumor immunogenicity by increasing the expression of tumor-associated antigens and increasing the level of chemokines in the tumor microenvironment. CD8 + T cells and dendritic cells are subsequently accelerated to be recruited and activated to induce anti-tumor immune effects in the tumor site.^6^ Immune checkpoint inhibitors (ICIs) not only activate CD8 + T cells but also normalizes the tumor vasculature system to reduce the tumor hypoxia state and increase tumor radiosensitivity.^7^ However, the efficacy of this combination is influenced by multiple factors, including the therapeutic sequence and the use of combination drugs. In terms of therapeutical sequence, concurrent therapies (within a 4-week interval) generally show better efficacy than sequential therapies.^8^ Nonetheless, while the combination therapy has improved the control of intracranial lesions, it has concurrently escalated the prevalence of treatment-related adverse reactions, such as radiation encephalitis, immune-related adverse effect and chemotherapy-induced nausea, and vomiting. Such side effects complicate the management of the disease and may necessitate an increased reliance on glucocorticoids (GCs) throughout the treatment period.

GCs, as frequently used adjuvant drugs in patients with brain metastases, can alleviate tumor-related symptoms (fatigue, dyspnea, intestinal obstruction and intracranial edema), tumor-unrelated comorbidities (autoimmune disorders, exacerbation of COPD, and prevention of hypersensitivity reactions), and adverse effects related to radiation, chemotherapy, and immunotherapy.^9^ Peritumoral brain edema (PTBE) is a major cause of morbidity and mortality in patients with brain tumors. Disruption of the structural integrity of the blood–brain barrier (BBB) and subsequent increase in interstitial fluid leads to this type of brain edema, termed vasogenic brain edema (VBE). VBE mediated by VEGF and other inflammatory mediators results in disruption of the BBB from functional loss of tight junction proteins.^10^ GCs can reverse VBE by regulating the expression of tight junction proteins, including occludin, claudin-5, claudin-1, and ZO-1 to relieve neurological symptoms.^11^ GCs have a dose-dependent immunosuppressive effect on both innate and adaptive immunity, which may impair the activation, expansion, and recruitment of T regulatory cells.^12,13^ Several studies have indicated that GCs use negatively impacts the efficacy of immune checkpoint inhibitors.^14^ Although GCs may improve the quality of life for patients with brain metastases by effectively controlling adverse effects, it is unknown whether various GC doses impair the survival of patients receiving immunotherapy combined with intracranial radiotherapy, as these patients are often administered high doses of GCs in clinical practice. Previous studies on the impact of GCs on the efficacy of immunotherapy have demonstrated that early steroid use impairs the adaptive immune response augmented by immunotherapy through reducing the peripheral pool of T lymphocytes, ultimately diminishing clinical efficacy.^15^ Once anti-tumor immunity is initiated, the negative effects of GCs on immune activation are significantly reduced. In several retrospective studies, baseline use of > 10 mg/day prednisone or its equivalents at the initiation of immunotherapy has been shown to detrimentally affect the objective response rate (ORR), overall survival (OS), and progression-free survival (PFS) in patients with advanced NSCLC receiving immunotherapy.^16–18^

This single-armed study focuses on the patients with NSCLC brain metastases treated by immunotherapy combined with intracranial radiotherapy. In our study, the overall doses of GCs for each patient were expressed as the cumulative total dose of dexamethasone equivalents during the baseline period of immunotherapy (28 days before and after the first immunotherapy). We analyze the influence of various baseline GC doses on the control of intracranial lesions and survival outcomes to provide better guidance for balancing adverse effect management and efficacy inhibition.

## Methods

### Patients

We collected data from 62 patients with NSCLC brain metastases who received immunotherapy combined with intracranial radiotherapy within 28 days of each other (the interval between immunotherapy and brain radiotherapy is less than 28 days^8^) between January 2020 and December 2022. Patients were from two institutions, including the First Affiliated Hospital of Chongqing Medical University and the Second Affiliated Hospital of Chongqing Medical University. All patients had received GCs during concurrent therapy. The researchers reviewed the patients’ medical records and confirmed the overall doses of GCs for each patient received during the baseline period of immunotherapy (28 days before and after the first immunotherapy^16,19^). Overall doses of GCs for each patient were expressed as cumulative total milligrams of dexamethasone equivalents over the period. The main types of GCs received by patients were dexamethasone and prednisone. Dexamethasone was commonly administered intravenously for the control of brain edema caused by brain metastasis, while prednisone was commonly administered orally. The dose conversion ratio of dexamethasone to prednisone is 0.75:5. As for the GCs administration regimen for each patient, the initial daily dose of dexamethasone was usually 5 mg/d or 10 mg/d, and was often reduced or discontinued within 1 to 2 weeks based on the severity of the patient’s symptoms related to cerebral edema, which was under the guidance of the congress of neurological surgeons systematic review and evidence-based guidelines for the treatment of adults with metastatic brain tumors.^20^ Prednisone was often taken orally by patients before chemotherapy to prevent chemotherapy-related allergic reactions with a daily dosage of 5–8 mg/d for 3 days. Platinum-based chemotherapies were the commonly used chemotherapy regimens, including albumin paclitaxel combined with platinum and pemetrexed combined with platinum. Patients receiving pemetrexed often took prednisone orally to prevent chemotherapy-related allergic symptoms. The clinicopathological characteristics of all patients were also collected, including age, sex, histology, Eastern Oncology Cooperative Group Physical Status (ECOG PS), smoking history, brain metastases status, systematic treatment schemes, and baseline hematological indices. PTBE staging for each patient was assessed by Edema Index (EI), which represents the value of the volume of edema and tumor divided by the tumor volume. (EI = 1: none; 1 < EI < 1.5: Light; 1.5 ≤ EI < 3: Moderate; ≥ 3: Severe).^21^ The Response Evaluation Criteria in Solid Tumors version 1.1 (RECIST 1.1) was used to assess the therapeutical efficacy, including complete response (CR), partial response (PR), stable disease (SD), and progressive disease (PD). Response assessment in neuro-oncology-brain metastasis (RANO-BM) criteria was used for assessment of intracranial lesions and intracranial response to the combination therapy. This study was approved by the Ethics Committee of the Second Affiliated Hospital of Chongqing Medical University and complied with the relevant requirements of the Declaration of Helsinki (1964).

### Statistical Analysis

This study summarized the characteristics of patients by means of descriptive statistics. To further explore the impact of baseline clinicopathological factors on the survival outcomes, a cox proportional hazards model was used for univariate analysis to assess the effect of variables on intracranial progression-free survival (iPFS), PFS, and OS. Variables that were significantly correlated were subsequently included in the multivariate model. The results of the Cox proportional hazards model analysis were reported as hazard ratios (HR) and 95% confidence intervals (CI). Standard propensity score matching (PSM) was employed to balance the variables that were significantly different at baseline across different GC dose stratification groups. Statistical analyses were performed using SPSS 27.0 software, and survival curves were plotted by GraphPad Prism 8.0. The statistical significance threshold for all statistical tests and survival analyses was set at *P* < .05.

### Outcomes

Long-term survival outcomes and short-term therapeutical effects were two aspects that we mainly analyzed. For long-term survival outcomes, OS was defined as the months from the initiation of concurrent therapy to the patient’s death from any cause, PFS was defined as the months from the initiation of concurrent therapy to the patient’s death or disease progression from any cause and iPFS was defined as the months from the initiation of concurrent therapy to the patient’s death from any cause or intracranial progression. Survival curves were plotted by the Kaplan–Meier method and compared by the log-rank test. Short-term therapeutical effects including intracranial objective response rate (iORR) and ORR were assessed at the time after intracranial radiotherapy and two cycles of immunotherapy, best intracranial objective response rate (BiORR) and best objective response rate (BORR) were assessed based on the best remission state. The χ^2^ test or Fisher’s exact test was used to analyze the effects of different dose stratification of GCs on iORR, ORR, BiORR, and BORR.

## Results

### Patient Characteristics

We identified 62 patients with NSCLC brain metastases who received immunotherapy combined with intracranial radiotherapy. In this study, we observed that patients all received the PD-1 immune checkpoint inhibitors, including Sintilimab, Camrelizumab, or Tislelizumab. The median age of these patients was 64.5 years, 82.3% were male, and 79.1% had non-squamous NSCLC. Additionally, 59.7% had a history of smoking, 58.1% had an ECOG PS score of 0 or 1, 69.4% exhibited symptomatic brain metastases, 59.7% had a Graded Prognostic Assessment (GPA) score of less than 2.5, and 59.6% were assessed as having moderate or severe intracranial edema. The overall GCs doses used by patients in this study were expressed as the total dexamethasone equivalent dose, with a median of 79 mg (interquartile spacing from 30.0 mg to 141.3 mg). The main indications for GCs application in patients were treatment for symptoms caused by brain edema (77.4%), pretreatment prior to chemotherapy for the prevention of allergy or vomiting (33.9%), and other indications for the alleviation of symptoms such as wheezing and vomiting (3.2%). The overall dose of GCs that patients received as pretreatment before chemotherapy was usually less than 30 mg. Patients with brain edema often received GCs at doses of more than 30 mg, with a median dose of 100mg for those receiving more than 30 mg. Therefore, we divided the patients into three groups according to the purpose of GCs administration. 27.4% of patients received GCs doses less than 30 mg, defined as low GCs use, 32.3% received GCs doses greater than or equal to 30 mg and less than 100 mg defined as medium GCs use, and 40.3% received GCs doses of 100 mg or more, defined as high GCs use. Table 1 summarizes the baseline characteristics of all the patients.

**Table 1.** Demographics and Baseline Characteristics of Patients Included

Characteristics	*N* (%), total *N* = 62	Characteristics	*N* (%), total *N* = 62
**Age,** median range	64.5 51.0–81.0	**Number of brain metastases**	
<65	31 (50.0)	≤3	15 (24.2)
≥65	31 (50.0)	>3	47 (75.8)
**Gender**		**Maximum diameter of brain metastases (cm)**	
Male	51 (82.3)	<2	27 (43.5)
Female	11 (17.7)	≥2	35 (56.5)
**Smoking history**		**Sites of brain metastases**	
Yes	37 (59.7)	Frontal lobe	28 (45.1)
No	25 (40.3)	Parietal lobe	22 (35.5)
**ECOG PS**		Occipital lobe	15 (24.2)
0	13 (21.0)	Temporal lobe	10 (16.3)
1	23 (37.1)	Cerebellum	15 (24.2)
2	21 (33.9)	Brainstem	2 (3.2)
3	5 (8.1)		
**Pathological pattern**		**PTBE staging**	
Adenocarcinoma	47 (75.8)	None	6 (9.7)
Squamous	13 (21.0)	Light	19 (30.6)
Others	2 (3.3)	Moderate	34 (54.8)
**Symptomatic brain metastases**		Severe	3 (4.8)
Yes	43 (69.4)	**GPA score**	
No	19 (30.6)	<2.5	37 (59.7)
**Sites of extracranial metastases**		≥2.5	25 (40.3)
Bone	24 (38.7)		
Adrenal	11 (17.7)	**Total GCs intake (mg)**	
Lung	9 (14.5)	Median	79.00
Liver Others	8 (12.9) 2 (3.3)	IQR	30.0–141.3
**Treatment line**		**Purpose of GCs use**	
1	40 (64.5)	Chemotherapy pretreatment	21 (33.9)
2	14 (22.6)	Brain edema therapy	48 (77.4)
≥3	8 (12.9)	Others	2 (3.2)
**Combined therapy**		**GCs intake grouping**	
Chemotherapy	50 (80.6)	GCs < 30 mg	17 (27.4)
Antiangiogenic therapy	24 (38.7)	30 mg ≤ GCs < 100 mg	20 (32.3)
**Radiotherapy mode**		GCs ≥ 100 mg	25 (40.3)
WBRT	41 (66.1)		
WBRT + SIB	4 (6.5)		
SRS	17 (27.4)		

Abbreviations: ECOG PS, Eastern Cooperative Oncology Group Performance Status; WBRT, whole brain radiation therapy; SIB, sequential integrated boost; SRS, stereotactic radiotherapy; GPA, Graded Prognostic Assessment; PTBE, peritumoral brain edema; IQR, interquartile range.

*PTBE staging was assessed by Edema Index (EI) which represents the value of the volume of edema and tumor divided by the tumor volume. (EI = 1: none; 1 < EI < 1.5: Light; 1.5 ≤ EI < 3: Moderate; ≥ 3: Severe).

### Impact of Steroid Use on Clinical Outcomes

**Figure 1.**
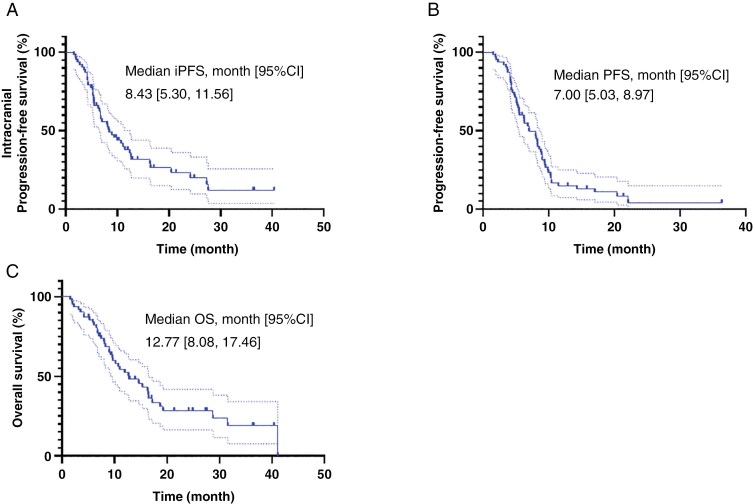
Kaplan–Meier curves of the overall population receiving GCs for (A) Intracranial progression-free survival (iPFS); (B) Progression-free survival (PFS); (C) Overall survival (OS).

We firstly analyzed the survival outcomes in the overall population to assess the impact of GCs on the overall subjects we researched. The median iPFS of the entire cohort was 8.43 months (95% CI 5.30–11.56) (Figure 1A); the median PFS of the overall population was 7.00 months (95% CI 5.03–8.97) (Figure 1B); the median OS of the overall population was 12.77 months (95% CI 8.08–17.46) (Figure 1C).

**Figure 2.**
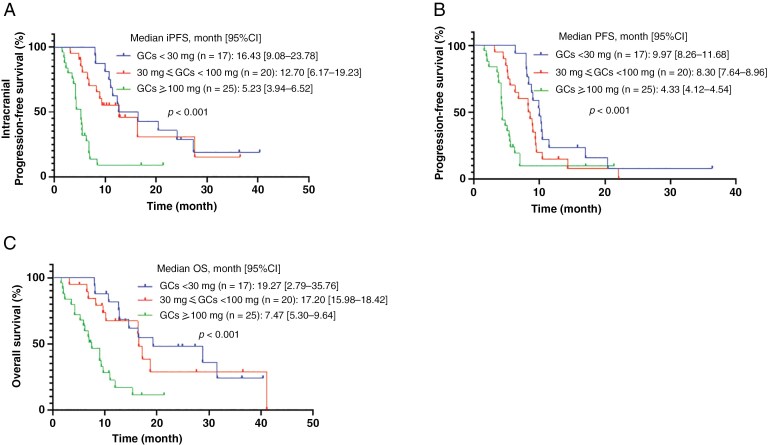
Kaplan–Meier curves for (A) Intracranial progression-free survival (iPFS); (B) Progression-free survival (PFS); (C) Overall survival (OS) according to the grouping of steroids dosage.

The subgroup analyses were then further explored among the three groups of patients receiving different GC doses. The median iPFS of patients in the high GCs use group was significantly worse than that in the medium GCs use group (5.23 months vs. 12.70 months; HR = 3.185, 95% CI 1.585–6.402, *P* < .001) and also worse than that in the low GCs use group (5.23 months vs. 16.43 months; HR = 3.602, 95% CI 1.769–7.333, *P* = .001). However, no significant difference was observed between the low and medium GCs use groups in their median iPFS (Figure 2A). Similarly, the median PFS of patients in the high GCs use group of was significantly worse than that in the medium GCs use group (4.33 months vs. 8.30 months; HR = 2.103, 95% CI 1.109–3.987, *P* = .011), and also worse than that in the low GCs use group (4.33 months vs. 9.97 months; HR = 2.831, 95% CI 1.435–5.586, *P* = .001). No significant difference was observed in median PFS between the low and medium GCs use groups (Figure 2B). Similar trends were observed for median OS among the three groups (Figure 2C).

The impact of baseline clinicopathological factors on the survival outcomes in iPFS, PFS, and OS were presented as below. In the cox multivariate model, GPA score and total GCs intake (HR = 1.016, 95% CI 1.008–1.024, *P* < .001) were independently associated with worse iPFS (Table 2). GPA score, maximum diameter of brain metastases, and total GCs intake (HR = 1.015, 95% CI 1.008–1.023, *P* < .001) were independently associated with worse PFS (Supplementary Table 1). The presence of extracranial metastases and total GCs intake (HR = 1.008, 95% CI 1.001–1.015, *P* = .030) were independently associated with worse OS (Supplementary Table 2).

**Table 2.** Cox Regression Analysis of iPFS (*N* = 62, 46 progression events)

	Univariable analysis		Multivariable analysis	
	HR (95% CI)	*P*	HR (95% CI)	*P*
**Demographic**				
Male (vs female)	0.708 (0.338–1.482)	0.360		
Age (continuous)	1.025 (0.985–1.068)	0.227		
ECOG PS	1.449 (1.075–1.953)	**0.015**	1.018 (0.667–1.556)	0.933
Smoking history	1.073 (0.584–1.971)	0.820		
**GPA score ≥ 2.5** **(reference < 2.5)**	0.419 (0.225–0.781)	**0.006**	0.408 (0.192–0.866)	**0.020**
**Tumor characteristics**				
Adenocarcinoma (vs others)	0.817 (0.402–1.662)	0.577		
Squamous cell carcinoma (vs others)	1.224 (0.602–2.489)	0.652		
**Brain mets** **characteristics**				
Extracranial mets (vs none)	2.007 (1.073–3.753)	**0.029**	1.133 (0.543–2.364)	0.739
Symptomatic brain mets (vs none)	2.591 (1.341–5.003)	**0.005**	1.986 (0.637–6.197)	0.237
Number of brain mets > 3 (reference ≤ 3)	0.717 (0.370–1.391)	0.325		
Maximum diameter of brain mets ≥ 2cm (reference < 2cm)	2.208 (1.210–4.028)	**0.010**	1.411 (0.682–2.922)	0.353
**Treatment line ≥ 2** (reference < 2)	0.822 (0.383–1.765)	0.616		
**WBRT (vs others)**	1.626 (0.859–3.078)	0.135		
**Combined systematic therapy**				
Chemotherapy (vs none)	0.632 (0.311–1.282)	0.203		
Antiangiogenic therapy (vs none)	1.072 (0.594–1.936)	0.817		
**PTBE (EI ≥ 1.5 vs < 1.5)**	2.219 (1.209–4.071)	**0.010**	0.327 (0.096–1.118)	0.075
**Hematologic index**				
CRP (continuous)	1.003 (0.999–1.007)	0.097		
LDH (continuous)	1.001 (0.999–1.002)	0.367		
NLR (continuous)	1.036 (1.011–1.062)	**0.005**	0.998 (0.945–1.054)	0.935
PLR (continuous)	1.002 (1.001–1.003)	**0.003**	1.001 (0.998–1.004)	0.534
**Total GCs intake (mg)**	1.012 (1.008–1.017)	**<0.001**	1.016 (1.008–1.024)	**<0.001**

**Figure 3.**
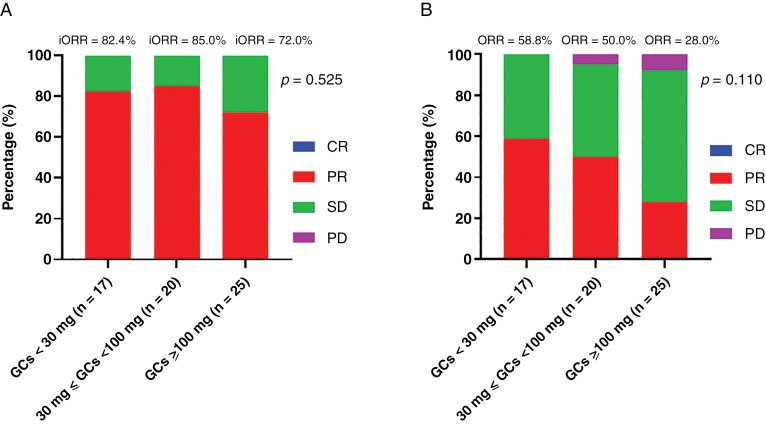
Objective response rates for (A) intracranial objective response rates (iORR); (B) objective response rates (ORR). CR, complete response; PR, partial response; SD, stable disease; PD, progressive disease.

We finally analyzed the short-term therapeutical effects among the three groups of patients receiving different GCs doses. No significant differences were observed in iORR (82.4% vs. 85.0% vs. 72.0%, *P* = .525) among the three groups (Figure 3A). Similarly, no significant differences were found in ORR (58.8% vs. 50.0% vs. 28.0%, *P* = .110) among the three groups (Figure 3B). No significant differences were observed in BiORR (88.2% vs. 85.0% vs. 76.0%, *P* = .552) and BORR (64.7% vs. 60.0% vs. 32.0%, *P* = .064) among the three groups.

### Propensity Score Matching Analysis

Based on the preliminary results, we observed that the median iPFS, median PFS, and median OS of the high GCs use group were significantly worse than those of the medium and low GCs use groups. Meanwhile, no significant differences in survival outcomes were observed between the medium and low GCs use groups. Therefore, we divided the overall patients into two groups using a GCs dose of 100 mg as the cut-off point for further investigation.

**Figure 4.**
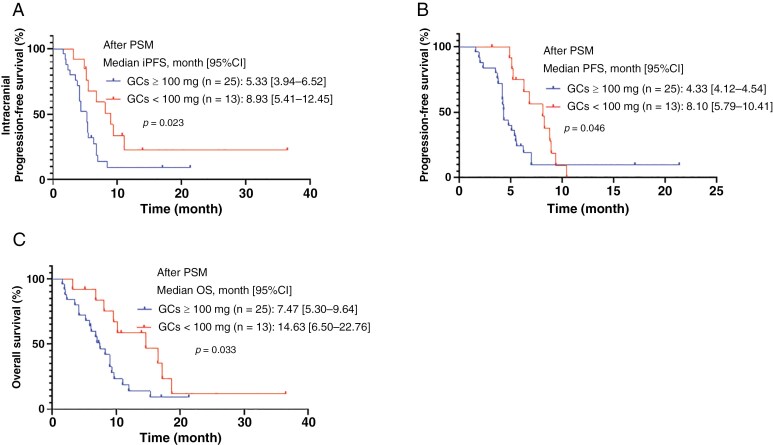
Kaplan–Meier curves for (A) intracranial progression-free survival (iPFS); (B) progression-free survival (PFS); (C) overall survival (OS) according to the grouping of steroids dosage after the propensity score matching (PSM).

Significant differences were found in ECOG PS score, GPA score, symptomatic brain metastases, maximum diameter of brain metastases, and degrees of brain edema between the two groups. Comparing to patients with low or medium GCs use, patients with high GCs use had more baseline clinicopathologic factors associated with poorer survival outcomes. It remains unclear whether the poorer survival outcomes of the high GCs use group are related to negative baseline prognostic factors or the inhibitory effect of GCs on ICIs. Therefore, propensity score matching was applied to balance these baseline differences (Supplementary Table 3). It was observed that the two balanced groups of patients still had significant differences in median iPFS (5.33 months vs. 8.93 months; HR = 2.308, 95% CI 1.141–4.668, *P* = .023) (Figure 4A), PFS (4.33 months vs. 8.10 months; HR = 2.007, 95% CI 1.015–3.972, *P* = .046) (Figure 4B), and OS (7.47 months vs. 14.63 months; HR = 2.229, 95% CI 1.089–4.560, *P* = .033) (Figure 4C).

## Discussion

Our study has revealed the relationship between poor prognosis and the use of high doses of GCs during immunotherapy combined with intracranial radiotherapy in patients with NSCLC brain metastases. The median iPFS, median PFS, and median OS of the patients in our study are comparable to those reported in previous studies on NSCLC brain metastases treated with immunotherapy combined with intracranial radiotherapy.^22–24^ Based on our research, we believe that high doses of GCs use may lead to worse survival outcomes in those patients, although there is a lack of effective evidence due to the absence of information on GCs use in previous studies. Furthermore, the median iPFS, median PFS of patients in our study are significantly longer than those patients who received single-agent immunotherapy.^25,26^ The patients who were receiving GCs at the initiation of single-agent immunotherapy were excluded in previous studies. It suggests that patients with NSCLC brain metastases can still gain a prognostic benefit from treatment with immunotherapy combined with intracranial radiotherapy, compared to single-agent immunotherapy, even though the use of GCs may negatively impact survival outcomes.

Patients in our study who received high doses of GCs had significantly poorer survival outcomes compared to those who received low or medium doses of GCs. We attribute this result to the inhibitory effect of GCs on the efficacy of ICIs, which becomes more pronounced with higher doses of GCs intake. The association between steroid use and decreased OS and PFS in patients that received steroids for supportive care or brain metastases has been confirmed by a large systematic review and meta-analysis.^27^ Worse PFS and OS were observed in patients receiving greater than 20 mg of prednisone in comparison with patients receiving less than 20 mg of prednisone in a study researched on patients with NSCLC treated with ICI monotherapy.^16^ Additionally, a multicentric retrospective analysis by Xue Bai et al.^28^ found that PFS and OS were worse among patients with malignant melanoma receiving high-dose GCs (≥ 60 mg prednisone equivalents once daily) for immune-related adverse events (irAEs) comparing with those who received lower doses of prednisone. These findings are consistent with the results of our study, which indicate that the baseline use of high doses of GCs impairs the PFS and OS, and higher doses of GCs have more pronounced negative impact. Furthermore, our study found that patients with NSCLC brain metastases receiving immunotherapy combined with intracranial radiotherapy were more liable to receive higher doses of GCs at baseline. Consequently, the survival outcome cut-point of GCs (100 mg dexamethasone equivalents) was much higher than that reported in previous studies.

In our study, no difference was observed in the short-term therapeutic responses among the three groups. Intracranial radiotherapy often plays a crucial role in the short-term control of intracranial lesions, whereas immunotherapeutic agents are more effective for long-term control of both intracranial lesions and extracranial lesions. Therefore, within the limited timeframe of assessing therapeutic responses of intracranial lesions, the negative impact of GCs on the efficacy of ICIs may be overshadowed by the positive effects of intracranial radiotherapy.

There are still some controversial conclusions about the influence of GCs use on the efficacy of ICIs in patients undergoing immunotherapy efficacy. The purposes of GCs can be divided into two categories: tumor-related indications (such as cerebral edema, dyspnea, and fatigue) and tumor-unrelated indications (such as irAEs, autoimmune diseases, and chemotherapy pretreatment). Several studies had found that the application of GCs prior to immunochemotherapy or for the treatment of irAEs in the late phase of immunotherapy did not significantly affect the survival outcomes of patients with NSCLC.^29,30^ In our study, patients in high GCs group receiving GCs mainly for the cerebral edema (tumor-related indication) had worse survival outcomes comparing with the patients in low GCs use group mainly for pretreatment prior to chemotherapy (non-tumor-related indication), which was consistent with the conclusion in the previous studies. A retrospective study by Ricciuti et al.^31^ discovered that the PFS and OS were longer in the non-tumor-associated steroids group comparing to the tumor-associated steroids group when treated with a baseline ≥ 10 mg of prednisone equivalents. Therefore, the purposes for which GCs are applied determine whether the efficacy of ICIs will be hampered, with only the GCs applied for cancer-related symptoms being associated with poorer survival outcomes. Routine use of dexamethasone for preventing chemotherapy or immunotherapy-associated adverse events should be still recommended for patients undergoing combined immunotherapy and chemotherapy.

Patients with poor ECOG PS or symptomatic brain metastases had been found to be more likely to receive GCs therapy in previous studies,^16,32^ which was in accordance with our results. The reason why such patients received GCs as supportive therapeutic agents may be due to their rapid clinical deterioration caused by these clinicopathologic factors. After balancing these baseline differences through PSM, we found that survival outcomes remained significantly different between the patients with high GCs use and those with medium or low GCs use. This illustrates that GCs still exert an inhibitory effect on the efficacy of immunotherapy combined with intracranial radiotherapy.

We found that there was no unified consensus in previous studies on GCs for the description and analysis of GCs doses. The purposes of some studies were aiming to explore whether baseline GCs were used could influence the efficacy of immunotherapy, and the definition for the use of GCs was above 10 mg/d prednisone equivalent,^16^ as this dose was deemed as the maximum daily physiological dose and adopted as the exclusion criterion in most clinical trials.^33,34^ When the study population received a relatively larger doses of GCs for the control of irAEs or received GCs continuously for multiple days, the cumulative doses of GCs used during the baseline period or the peak dose on a single day were usually calculated and analyzed. For example, whether the GCs were continuously or intermittently used and the baseline cumulative GCs doses were selected for analysis in one study researching on population receiving prednisone for consecutive days.^35^ Another study focused on the peak dose of GCs and the baseline cumulative GCs when concentrating on patients with irAEs.^36^ In another study exploring the effect of GCs application on immunotherapy before immunotherapy combined with chemotherapy, the average doses of dexamethasone used before each cycle of chemotherapy was selected for stratified analysis.^37^ In our study, the statistics method of cumulative total dose of dexamethasone equivalents was chosen for the following reasons: firstly, the research population was prone to receive different doses of GCs within which dexamethasone being the main type. Therefore, using the equivalent conversion based on dexamethasone dosage is more intuitive and convenient. Secondly, patients often received different doses of dexamethasone for multiple consecutive days to alleviate the symptoms caused by brain metastases, which was similar with the treatment mode of GCs on irAEs except for the GCs type. Due to the dose-dependent immunosuppressive effect of dexamethasone,^38^ we considered that it could be better to conduct the further statistical analysis and present the results by calculating the baseline cumulative dexamethasone equivalent doses instead of the expression of daily dosage in the study.

There were some limitations in our study. First, this study was retrospective with limited overall patient size. Second, this study did not evaluate the quality of life of the patients after the application of GCs. Third, the study only focused on the patients receiving the GCs at the baseline of immunotherapy, so the influence of GCs on immunotherapy during subsequent cycles of immunotherapy was not assessed. Fourth, drugs such as metformin and antibiotics, which might also impact the efficacy of immunotherapy, were not included and analyzed in this study.

Immunotherapy combined with intracranial radiotherapy had become an important therapeutic approach for patients with NSCLC brain metastases, yet the impact of adjunctive medications on the treatment is often ignored. In this study, we investigated the relationship between the high dose use of GCs and poor prognosis in patients with NSCLC brain metastases during immunotherapy combined with intracranial radiotherapy, confirming that baseline high dose GCs use (≥ 100 mg) reduces the efficacy of the combination therapy. Therefore, optimizing the use of anti-edema drugs such as mannitol, bevacizumab, diuretics, or hypertonic saline in combination with brain radiotherapy and immunotherapy could be considered to mitigate the adverse effects of GCs on the efficacy of ICIs.

## Supplementary Material

Supplementary material is available online at *Neuro-Oncology Advances* (https://academic.oup.com/noa).

vdaf158_suppl_Supplementary_Tables_S1-S3

## Data Availability

The data supporting the findings of this study will be available upon reasonable written request approved by the corresponding author.
