# Supplementary material for: Impact of baseline glucocorticoid use on the efficacy of immunotherapy combined with intracranial radiotherapy in NSCLC patients with brain metastases
Source: Neurooncol Adv. 2025 Jul 12;7(1):vdaf158. doi: 10.1093/noajnl/vdaf158 (PMC12311932; doi:10.1093/noajnl/vdaf158)
Supplement: vdaf158_suppl_Supplementary_Tables_S1-S3 [file vdaf158_suppl_supplementary_tables_s1-s3.docx]

**Supplementary Table 1**. Cox regression analysis of PFS (N= 62, 56 progression events)

|  | univariable analysis | |  | multivariable analysis | |
| --- | --- | --- | --- | --- | --- |
|  | HR (95% CI) | *p* |  | HR (95% CI) | *p* |
| **Demographic** |  |  |  |  |  |
| Male (vs female) | 0.685 (0.351-1.337) | 0.267 |  |  |  |
| Age (continuous) | 1.019 (0.983-1.057) | 0.306 |  |  |  |
| ECOG PS | 1.479 (1.135-1.926) | **0.004** |  | 1.079 (0.739-1.575) | 0.693 |
| Smoking history | 1.108 (0.644-1.907) | 0.710 |  |  |  |
| **GPA score≥2.5**  **(reference<2.5)** | 0.376 (0.210-0.673) | **<0.001** |  | 0.418 (0.202-0.868) | **0.019** |
| **Tumor characteristics** |  |  |  |  |  |
| Adenocarcinoma (vs  others) | 0.787 (0.405-1.531) | 0.311 |  |  |  |
| Squamous cell  carcinoma (vs others) | 1.270 (0.653-2.470) | 0.481 |  |  |  |
| **Brain mets**  **characteristics** |  |  |  |  |  |
| Extracranial mets (vs  none) | 1.800 (1.020-1.174) | **0.042** |  | 0.798 (0.400-1.592) | 0.522 |
| Symptomatic brain mets (vs none) | 2.607 (1.448-4.692) | **0.001** |  | 2.394 (0.836-6.854) | 0.104 |
| Number of brain mets>3 (reference≤3) | 0.436 (0.233-0.816) | **0.009** |  | 0.602 (0.301-1.208) | 0.153 |
| Maximum diameter of  brain mets≥2cm  (reference<2cm) | 3.529 (1.947-6.396) | **<0.001** |  | 2.590 (1.259-5.327) | **0.010** |
| **Treatment line≥2**  (reference<2) | 0.846 (0.481-1.488) | 0.561 |  |  |  |
| **WBRT (vs others)** | 1.165 (0.661-2.054) | 0.597 |  |  |  |
| **Combined systematic therapy** |  |  |  |  |  |
| Chemotherapy (vs  none) | 0.661 (0.340-1.285) | 0.222 |  |  |  |
| Antiangiogenic therapy (vs none) | 0.819 (0.475-1.413) | 0.474 |  |  |  |
| **PTBE (EI≥1.5 vs <1.5)** | 2.166 (1.256-3.735) | **0.005** |  | 0.343 (0.117-1.002) | 0.056 |
| **Hematologic index** |  |  |  |  |  |
| CRP (continuous) | 1.003 (1.000-1.007) | 0.087 |  |  |  |
| LDH (continuous) | 1.001 (0.999-1.003) | 0.511 |  |  |  |
| NLR (continuous) | 1.039 (1.015-1.063) | **0.001** |  | 1.035 (0.986-1.087) | 0.164 |
| PLR (continuous) | 1.002 (1.001-1.003) | **0.008** |  | 0.999 (0.996-1.001) | 0.333 |
| **Total GCs intake (mg)** | 1.011 (1.007-1.015) | **<0.001** |  | 1.015 (1.008-1.023) | **<0.001** |

**Supplementary Table 2**. Cox regression analysis of OS (N= 62, 39 progression events)

|  | univariable analysis | |  | multivariable analysis | |
| --- | --- | --- | --- | --- | --- |
|  | HR (95% CI) | *p* |  | HR (95% CI) | *p* |
| **Demographic** |  |  |  |  |  |
| Male (vs female) | 0.883 (0.390-2.001) | 0.765 |  |  |  |
| Age (continuous) | 1.015 (0.971-1.062) | 0.504 |  |  |  |
| ECOG PS | 1.612 (1.169-2.225) | **0.004** |  | 1.018 (0.613-1.690) | 0.946 |
| Smoking history | 1.041 (0.547-1.981) | 0.903 |  |  |  |
| **GPA score≥2.5**  **(reference<2.5)** | 0.405 (0.202-0.812) | **0.011** |  | 0.605 (0.246-1.488) | 0.274 |
| **Tumor characteristics** |  |  |  |  |  |
| Adenocarcinoma (vs  others) | 0.788 (0.371-1.671) | 0.534 |  |  |  |
| Squamous cell  carcinoma (vs others) | 1.270 (0.599-2.693) | 0.412 |  |  |  |
| **Brain mets**  **characteristics** |  |  |  |  |  |
| Extracranial mets (vs  none) | 2.898 (1.436-5.850) | **0.003** |  | 2.556 (1.126-5.801) | **0.025** |
| Symptomatic brain mets (vs none) | 3.251 (1.523-6.941) | **0.002** |  | 1.619 (0.378-6.930) | 0.516 |
| Number of brain mets>3 (reference≤3) | 0.757 (0.367-1.562) | 0.452 |  |  |  |
| Maximum diameter of  brain mets≥2cm  (reference<2cm) | 2.132 (1.120-4.056) | **0.021** |  | 0.958 (0.410-2.237) | 0.921 |
| **Treatment line≥2**  (reference<2) | 0.867 (0.449-1.675) | 0.671 |  |  |  |
| **WBRT (vs others)** | 2.148 (1.043-4.423) | **0.038** |  | 2.198 (0.891-5.418) | 0.087 |
| **Combined systematic therapy** |  |  |  |  |  |
| Chemotherapy (vs  none) | 0.550 (0.260-1.166) | 0.119 |  |  |  |
| Antiangiogenic therapy (vs none) | 1.015 (0.537-1.919) | 0.962 |  |  |  |
| **PTBE (EI≥1.5 vs <1.5)** | 2.843 (1.449-5.580) | **0.002** |  | 1.563 (0.434-5.628) | 0.495 |
| **Hematologic index** |  |  |  |  |  |
| CRP (continuous) | 1.003 (1.000-1.007) | 0.196 |  |  |  |
| LDH (continuous) | 1.001 (0.999-1.003) | 0.480 |  |  |  |
| NLR (continuous) | 1.046 (1.020-1.072) | **<0.001** |  | 1.053 (0.986-1.124) | 0.123 |
| PLR (continuous) | 1.002 (1.001-1.004) | **0.001** |  | 0.998 (0.995-1.002) | 0.397 |
| **Total GCs intake (mg)** | 1.012 (1.006-1.016) | **<0.001** |  | 1.008 (1.001-1.015) | **0.030** |

**Supplementary Table 3.** Patient characteristics in the full cohort of patients and in the propensity score matching sample

|  | General population | | | |  | | Propensity score matched groups | | |
| --- | --- | --- | --- | --- | --- | --- | --- | --- | --- |
|  | GCs<100mg (n=37) | GCs≥100mg (n=25) | *p* |  | | GCs<100mg (n=13) | | GCs≥100mg (n=25) | *p* |
| Gender |  |  | 0.526 | |  | |  |  | 0.392 |
| Female | 8 (21.62%) | 3 (12.00%) |  | |  | | 3 (23.08%) | 3 (12%) |  |
| Male | 29 (78.38%) | 22 (88.00%) |  | |  | | 10 (76.92%) | 22 (88%) |  |
| Age | 63.38±6.92 | 65.48±8.54 | 0.290 | |  | | 64.62±6.99 | 65.48±8.54 | 0.755 |
| ECOG PS |  |  | 0.001 | |  | |  |  | 0.534 |
| 0 | 13 (35.14%) | 0 (0%) |  | |  | | 0 (0%) | 0 (0%) |  |
| 1 | 15 (40.54%) | 8 (32.00%) |  | |  | | 6 (46.15%) | 8 (32%) |  |
| 2 | 6 (16.22%) | 15 (60%) |  | |  | | 5 (38.46%) | 15 (60%) |  |
| 3 | 3 (8.11%) | 2 (8%) |  | |  | | 2 (15.38%) | 2 (8%) |  |
| Smoking history |  |  | 0.568 | |  | |  |  | 1.000 |
| No | 16 (43.24%) | 9 (36%) |  | |  | | 4 (30.77%) | 9 (36%) |  |
| Yes | 21 (56.76%) | 16 (64%) |  | |  | | 9 (69.23%) | 16 (64%) |  |
| GPA score |  |  | 0.031 | |  | |  |  | 1.000 |
| <2.5 | 18 (48.65%) | 19 (76%) |  | |  | | 10 (76.92%) | 19 (76%) |  |
| ≥2.5 | 19 (51.35%) | 6 (24%) |  | |  | | 3 (23.08%) | 6 (24%) |  |
| Adenocarcinoma |  |  | 0.565 | |  | |  |  | 1.000 |
| No | 8 (21.62%) | 7 (28%) |  | |  | | 3 (23.08%) | 7 (28%) |  |
| Yes | 29 (78.38%) | 18 (72%) |  | |  | | 10 (76.92%) | 18 (72%) |  |
| Squamous cell carcinoma |  |  | 0.630 | |  | |  |  | 1.000 |
| No | 30 (81.08%) | 19 (76%) |  | |  | | 10 (76.92%) | 19 (76%) |  |
| Yes | 7 (18.92%) | 6 (24%) |  | |  | | 3 (23.08%) | 6 (24%) |  |
| Extracranial mets |  |  | 0.155 | |  | |  |  | 0.714 |
| No | 17 (45.95%) | 7 (28%) |  | |  | | 5 (38.46%) | 7 (28%) |  |
| Yes | 20 (54.05%) | 18 (72%) |  | |  | | 8 (61.54%) | 18 (72%) |  |
| Symptomatic brain mets |  |  | <0.001 | |  | |  |  | 1.000 |
| No | 19 (51.35%) | 0 (0%) |  | |  | | 0 (0%) | 0 (0%) |  |
| Yes | 18 (48.65%) | 25 (100%) |  | |  | | 13 (100%) | 25 (100%) |  |
| Number of BM |  |  | 0.565 | |  | |  |  | 1.000 |
| ≤3 | 29 (78.38%) | 18 (72%) |  | |  | | 9 (69.23%) | 18 (72%) |  |
| >3 | 8 (21.62%) | 7 (28%) |  | |  | | 4 (30.77%) | 7 (28%) |  |
| Maximum diameter of brain mets |  |  | 0.042 | |  | |  |  | 0.714 |
| <2cm | 17 (45.95%) | 18 (72%) |  | |  | | 8 (61.54%) | 18 (72%) |  |
| ≥2cm | 20 (54.05%) | 7 (28%) |  | |  | | 5 (38.46%) | 7 (28%) |  |
| Treatment line |  |  | 0.249 | |  | |  |  | 0.148 |
| <2 | 11 (29.73%) | 11 (44%) |  | |  | | 2 (15.38%) | 11 (44%) |  |
| ≥2 | 26 (70.27%) | 14 (56%) |  | |  | | 11 (84.62%) | 14 (56%) |  |
| Chemotherapy |  |  | 0.276 | |  | |  |  | 1.000 |
| No | 5 (13.51%) | 7 (28%) |  | |  | | 3 (23.08%) | 7 (28%) |  |
| Yes | 32 (86.49%) | 18 (72%) |  | |  | | 10 (76.92%) | 18 (72%) |  |
| Antiangiogenic therapy |  |  | 0.373 | |  | |  |  | 0.730 |
| No | 21 (56.76%) | 17 (68%) |  | |  | | 8 (61.54%) | 17 (68%) |  |
| Yes | 16 (43.24%) | 8 (32%) |  | |  | | 5 (38.46%) | 8 (32%) |  |
| WBRT |  |  | 0.798 | |  | |  |  | 0.714 |
| No | 13 (35.14%) | 8 (32%) |  | |  | | 3 (23.08%) | 8 (32%) |  |
| Yes | 24 (64.86%) | 17 (68%) |  | |  | | 10 (76.92%) | 17 (68%) |  |
| PTBE |  |  | <0.001 | |  | |  |  | 0.315 |
| <1.5 | 24 (64.86%) | 2 (8%) |  | |  | | 3 (23.08%) | 2 (8%) |  |
| ≥1.5 | 13 (35.14%) | 23 (92%) |  | |  | | 10 (76.92%) | 23 (92%) |  |
